# Salience-based object prioritization during active viewing of naturalistic scenes in young and older adults

**DOI:** 10.1038/s41598-020-78203-7

**Published:** 2020-12-16

**Authors:** Antje Nuthmann, Immo Schütz, Wolfgang Einhäuser

**Affiliations:** 1 Institute of Psychology, Kiel University, Olshausenstr. 62, 24118 Kiel, Germany; 2 School of Philosophy, Psychology and Language Sciences, University of Edinburgh, Edinburgh, UK; 3 Institute of Physics, Chemnitz University of Technology, Chemnitz, Germany; 4 Present Address: Justus Liebig University Giessen, Giessen, Germany

**Keywords:** Cognitive ageing, Attention, Perception

## Abstract

Whether fixation selection in real-world scenes is guided by image salience or by objects has been a matter of scientific debate. To contrast the two views, we compared effects of location-based and object-based visual salience in young and older (65 + years) adults. Generalized linear mixed models were used to assess the unique contribution of salience to fixation selection in scenes. When analysing fixation guidance without recurrence to objects, visual salience predicted whether image patches were fixated or not. This effect was reduced for the elderly, replicating an earlier finding. When using objects as the unit of analysis, we found that highly salient objects were more frequently selected for fixation than objects with low visual salience. Interestingly, this effect was larger for older adults. We also analysed where viewers fixate within objects, once they are selected. A preferred viewing location close to the centre of the object was found for both age groups. The results support the view that objects are important units of saccadic selection. Reconciling the salience view with the object view, we suggest that visual salience contributes to prioritization among objects. Moreover, the data point towards an increasing relevance of object-bound information with increasing age.

## Introduction

Which factors guide where we direct our attention and gaze when viewing a naturalistic scene? In recent years, this question has sparked a scientific debate about whether fixation selection is based on image properties such as visual salience (hereafter: salience view) or information about objects within the scene (hereafter: object view). Here, we attempt to reconcile the two views by comparing effects of location-based and object-based visual salience, utilizing statistical analyses that allow for assessing the unique contribution of salience to fixation selection in scenes. In addition, we explore how eye guidance in scenes changes across the lifespan by comparing young and older (65 + years) adults.

Computational models that highlight the role of low-level image features propose that the eyes are mainly driven toward image regions that are visually salient. The *saliency map* algorithms^1,2^ have become the reference models in this regard. Visual salience and saliency maps attempt to define areas that “stand out” from the background, the idea being that these regions are looked at before others. The saliency map model and its descendants indeed predict human fixation selection in free-viewing tasks reasonably well^3,4^.

The alternative object view stipulates that objects form an important unit of saccadic selection in scene viewing. Empirical support for object-based selection was obtained by analysing fixation locations on scene images with respect to object boundaries. Nuthmann and Henderson^5^ found that the distribution of fixations within objects is well-described by a 2-dimensional Gaussian distribution. The distribution has a mean close to the centre of the object, quantifying the *preferred viewing location* (PVL). The basic finding that viewers prefer to send their eyes to the centre of objects has since been replicated^6–10^. Additional research has shown that the PVL is modulated by low-level visuomotor variables such as object size, the direction of the incoming saccade, and launch site distance^5,11^. For example, saccade landing positions are systematically shifted towards their launch site; that is, incoming saccades tend to undershoot the centre of the object^5^, which is consistent with the hypometric bias that has been observed for saccades to single targets in peripheral vision^12,13^. Object category and affordances also play a role^10,14^. Finally, the PVL was found to be preserved when observers’ central vision was strongly degraded^15^, demonstrating the importance of peripheral vision to fixation selection.

According to the object view, salience does not guide fixation selection directly but through a correlation between salient locations and objects. In this view, the saliency map predicts fixations because it provides a (coarse) estimate of object locations. Indeed, the original saliency map^2,16^ has little additional predictive power for fixation locations if object locations are known^17^. This result has been challenged on the basis of detailed analyses of an extended set of more recent salience models^18^. However, an object-based model that adequately considers the object-based PVL^5^ predicts fixations equally well as the best low-level salience models^19^. When scenes are experimentally manipulated to dissociate objects from regions with high low-level salience, the object-based model even outperforms such models^19^. Pursuing a similar approach as in Stoll et al.^19^, Borji and Tanner^10^ found that a weighted linear combination of the map generated by the Adaptive Whitening Saliency (AWS) model^20^ and a map of object boundaries (adjusted for higher probability of fixations around the object’s centre of mass) achieved significantly better gaze prediction than either model alone.

One advantage of salience maps is that they are image computable, which implies that they can be derived by exclusively using information contained in the current image. By comparison, in the aforementioned studies advocating the object view of fixation selection in scenes, the objects were labelled manually by human annotators^5,19^. This is not a principled limitation as computational models for object detection continue to improve, especially those based on deep neural networks (DNNs)^21^. Such models have indeed been successfully adapted for fixation prediction^22^. Moreover, in the modelling literature “proto-objects” have been suggested as an image computable alternative to “real” objects^23^. Although the precise definition of the term varies, in the modelling literature “proto-object” typically refers to entities that are potential objects based on their image-computable properties. Proto-object models can yield improvements to salience map predictions^23,24^. However, one test which we deem critical is typically missing in the evaluation of such models: do fixations of human observers within proto-objects show the PVL phenomenon? For the model by Walther and Koch^25^, in which proto-objects are a function of image salience, Nuthmann and Henderson^5^ showed that no PVL was found for proto-objects unless they overlapped with annotated real objects.

Although the object view is supported by experimental and modelling results, a critical question remains: once the objects are available for selection, how do observers decide which object, out of several candidate objects, to select for fixation? In previous research, we have argued that object-based visual salience contributes to such prioritization among objects^19^. With the present study we extend this research by pursuing a number of interrelated goals. First, we set out to replicate our previous findings in an independent sample of young adults with different images. Second, we compared effects of location-based and object-based visual salience. Third, we investigated the PVL for first fixations on objects in scenes. Moreover, we compared eye movements of young and older adults.

It is important to study how well research findings generalize from young to older adults. Most psychology research is based on studies with young adults, primarily undergraduate psychology students^26^. At the same time, Western societies have to deal with ageing populations and a considerable demographic redistribution. Basic visual and cognitive functions decline with advancing age^27–29^. Specifically, older age is associated with subtle reductions in visual abilities. These include reductions in visual acuity^30^, contrast sensitivity^31^, and visual fields^32^.

Research using the additional singleton paradigm has shown that visually salient stimuli can capture attention and trigger an eye movement toward their location reflexively, regardless of an observer’s intentions^33^. Studies that examined age-related changes in capture susceptibility found mixed results. While some studies found greater oculomotor capture in aging adults^34,35^, others did not^36^. Other research has investigated how eye-movement behaviour changes across the adult lifespan. In simple saccade-targeting tasks, older adults showed increased saccade latency^37,38^ while saccade accuracy was found to be preserved^39^ or to be reduced^40,41^. Healthy aging also affects eye movements during sentence reading^42^. Older adults make more and, on average, longer fixations^43^. Accuracy in saccade targeting appears to be relatively preserved, as suggested by analyses of the PVL for words in reading^44^.

Açik et al.^45^ were the first to investigate developmental changes during scene viewing by comparing eye movements from young adults, older adults, and children (mean age 7.6 years). During a 5-s viewing period, older adults made more saccades than both young adults and children, but with shorter amplitude. At the same time, all three age groups showed similar levels of explorative viewing behaviour. Importantly, the influence of low-level image features on fixation selection in scenes was found to decrease with increasing age. Subsequent studies revealed that image salience can predict fixation locations in young children^46^ and infants^47^. The data by Helo et al.^46^ suggest that image salience was a better predictor for children between two and six years old than for older children and adults.

In the present study, we set out to replicate Açik et al.’s^45^ results for local low-level image features on fixation selection in scenes. However, instead of individual features we used a composite measure of image salience (specifically, the AWS model), and a different analysis method. Going beyond previous research, we additionally explored whether there were different effects of object-based visual salience for young and older adults. For both types of analyses, we used generalized linear mixed modelling (GLMM). To assess effects of location-based visual salience, we combined GLMM with a-priori scene parcellation using a grid with equal-sized, square cells^48,49^. This analysis approach has a number of desirable properties; perhaps most importantly, we can explicitly model the central bias of fixation^50,51^ by including a separate central-bias predictor in the GLMM. This allows us to test whether location-based visual salience has an independent effect above and beyond what can be accounted for by observers’ tendency to look at the centre of scene images, where high-salience items oftentimes appear. In addition, we can investigate age-related differences. In another set of analyses, we extended this approach by using object-based scene parcellation instead of a grid. With this object-based GLMM approach, we can analyse the independent contribution of object-based visual salience and other object properties (size, eccentricity) to object prioritization for gaze guidance^19^, and how these effects depend on age group. We extended our open-source Python toolbox *GridFix*^49^ to include the data processing steps for the object-based analyses presented here.

In sum, our approach enables us to compare age-related changes in the effects of object salience on fixation guidance (object GLMM) to those of local, object-agnostic scene salience (grid GLMM). For older adults, location-based visual salience should have a smaller effect on fixation probability than for younger adults^45^. If the influence of visual salience on fixation guidance generally decreases with age, effects of object salience on object prioritization should decrease as well. Alternatively, if older observers rely more heavily on objects and high-level structure, effects of object-based and location-based scene salience should be differentially modulated by age.

## Results

**Figure 1:**
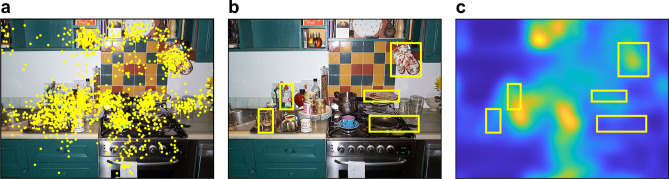
Real-world scene with all fixations from all 76 observers overlaid as yellow dots (**a**) and with tagged object bounding boxes represented by yellow rectangles (**b**). The salience map (Adaptive Whitening Saliency) for this image is shown in (**c**), where brighter colours correspond to higher visual salience; the object bounding boxes from (**b**) are additionally displayed. The depicted photograph was taken by George L. Malcolm.

Data from 42 young and 34 older adults were analysed. Every participant viewed 150 colour photographs of natural scenes (see Fig. 1 for an example) for 6 s each.

### Memory test performance and basic eye-movement measures

In a first step, we analysed participants’ responses to the memory test questions that occurred after 20% of the trials. The questions were yes/no questions that were related to objects in the scenes (e.g., “Was there an oven mitt?”). All observers had a positive *d*’, which is indicative of above-chance performance (young: *M* = 1.54, *SD* = 0.45, range: 0.67 to 2.33; old: *M* = 1.33, *SD* = 0.47, range: 0.27 to 2.13). There was no significant difference between the two age groups, *t*(69.8) = 1.97, *p* = 0.053. Observers in both groups applied conservative criteria *c*; that is, they had the tendency to rather classify a present item as absent (miss) than vice versa (false alarm). On average, older observers applied a more conservative criterion (*M* = 0.67, *SD* = 0.38, range: − 0.44 to 1.32) than young observers (*M* = 0.45, *SD* = 0.34, range: − 0.33 to 1.19), *t*(66.8) = 2.68, *p* = 0.009.

**Figure 2:**
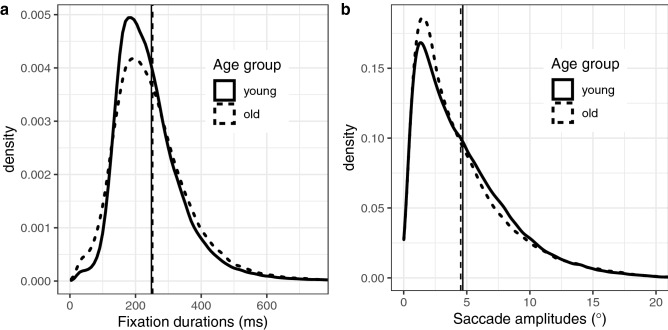
Basic eye-movement measures for young and older adults. Distributions of fixation durations (**a**) and saccade amplitudes (**b**) are shown. The vertical lines represent the corresponding means.

To characterize the eye-movement behaviour of young and older adults at a basic level, we calculated their mean number of fixations per trial, along with their mean fixation durations and saccade amplitudes (Table 1). There were no significant differences between the age groups on number of fixations (*t*(66.5) = 0.29, *p* = 0.773), fixation duration (*t*(57.18) = − 0.6, *p* = 0.553) or saccade amplitude (*t*(71.23) = 1.28, *p* = 0.204). However, the distributions of fixation durations and saccade amplitudes (Fig. 2) showed subtle differences between young and older adults, which suggests that there may be systematic differences in viewing behaviour which are not well captured by mean-level analyses.

**Table 1:** Mean (and standard deviation) general scanning behaviour per participant for young and older adults.

		Number of fixations per trial	Fixation duration (ms)	Saccade amplitude (°)
Age group	Young adults	21.03 (1.91)	248.39 (23.76)	4.68 (0.56)
	Older adults	20.9 (2.16)	252.49 (33.91)	4.52 (0.55)

### Effects of object-based visual salience on fixation selection

**Figure 3:**
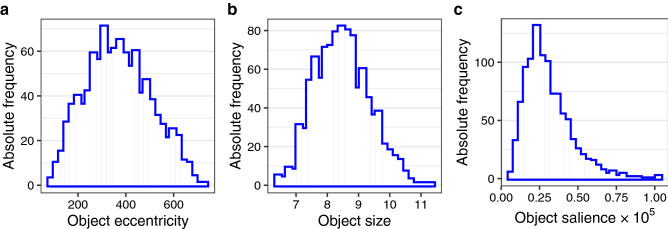
Distribution of object properties; (**a**) object eccentricity, (**b**) object size and (**c**) object salience.

The aim of the next analysis was to examine the effects of object eccentricity, size, and salience on fixation probability, and whether these effects differed between young and older adults. First, object eccentricity was included to account for observers’ central fixation bias. Based on our previous investigations^49^, an anisotropic Euclidean central-bias predictor was included in the GLMM (see “Methods” for details). Second, object size was defined as the log-transformed area (number of pixels) of the object’s bounding box (Fig. 1b). Third, object salience was defined as the mean over the normalized saliency map’s values within the object’s bounding box (Fig. 1c). Figure 3 shows the distribution of object properties for 1032 annotated objects (see “Methods” for additional details).

The three object-related input variables were measured on a continuous scale. Age group is a categorical variable, which was treatment-coded (reference category: young adults). Differences between young and older adults were tested through interactions between age group and a given continuous predictor. Thus, the GLMM included eight fixed effects (intercept, three main effects, four interaction coefficients).

**Figure 4:**
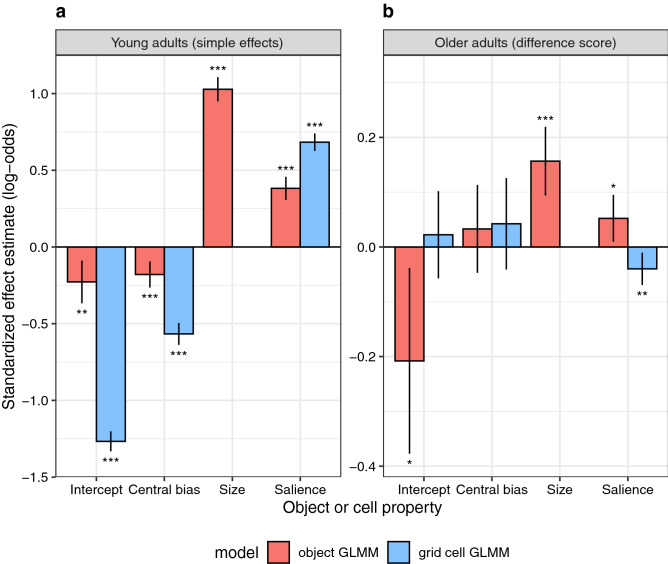
Fixed-effects results from the object GLMM (red bars) and the grid GLMM (blue bars), each fitting fixation probability during scene viewing for young (left) and older (right) adults. In particular, effects of object-based (red) and location-based (blue) visual salience on fixation probability are compared. (**a**) Effects that were estimated for the young adults. (**b**) Difference scores, describing the difference between older and young adults. Error bars indicate 95% confidence intervals. Stars denote coefficients that were significantly different from zero (* *p* < .05, ** *p* < .01, *** *p* < .001). Different to the object GLMM, the grid GLMM did not include a fixed-effect for *size* because all cells in the grid were of equal size.

The GLMM results are summarized in Table 2, and the fixed-effects estimates are visualized in Fig. 4 (red bars in both panels). Dependent variable is the probability of object fixation (1 yes, 0 no) in logit space. The intercept in the GLMM represents the overall fixation probability. Compared to the reference group of young adults (*b* = − 0.2278, *SE* = 0.071, *z* = − 3.207, *p* = 0.001), the model intercept was significantly lower for older adults (*b* = − 0.208, *SE* = 0.0865, *z* = − 2.406, *p* = 0.016). The fact that the intercept for the group of young adults is significantly different from zero has no interpretative meaning. The logit value of − 0.23 corresponds to a probability of 0.44. For the older adults, the actual coefficient for the intercept can be derived by summing the coefficient for young adults (− 0.2278) and the interaction coefficient (− 0.208). The interaction coefficient is a difference score, describing the difference between older and young adults. Converting the summed value to a probability value, it becomes clear that the overall fixation probability was reduced to 0.39 for the group of older adults. These seemingly low probability values for both participant groups do not imply that more than half of the annotated objects were never fixated; instead, these values indicate that not every participant fixated every object. The question then arises: what object properties determine whether some objects are prioritized over others?

**Table 2:** Object GLMM. Age-related effects on fixation probability during scene viewing are modelled relative to annotated objects in scenes.

Fixed effects	Description	*B*	*SE*	*z*	*p*
Intercept	Young	− 0.2278	0.071	− 3.207	0.001
Intercept	Old–young	− 0.208	0.0865	− 2.406	0.016
Central bias	Young	− 0.1795	0.0434	− 4.131	< 0.001
Central bias	Old–young	0.033	0.0409	0.807	0.420
Object size	Young	1.028	0.0403	25.485	< 0.001
Object size	Old–young	0.1567	0.032	4.901	< 0.001
Object salience	Young	0.3823	0.0384	9.949	< 0.001
Object salience	Old–young	0.0523	0.0219	2.387	0.017
**Random effects**					
Groups	Name	Variance	Correlation		
Subject	Intercept	0.13466	Intercept		
	Central bias	0.02521	0.73	Central bias	
	Size	0.01161	− 0.09	0.14	Size
	Salience	0.00288	0.12	− 0.28	0.17
Object	Intercept	1.03523	–	–	–
Scene	Intercept	0.09261	–	–	–

Standardized coefficients (*b*), standard errors (*SE*), *z*- and *p*-values for fixed effects and variances and correlations for random effects are provided.

Owing to the central bias, fixation probability was influenced by object eccentricity. Thus, young adults fixated centrally located objects more frequently than distant objects (*b* = − 0.1795, *S*E = 0.0434, *z* = − 4.131, *p* < 0.001), and this effect did not differ between age groups (*b* = 0.033, *SE* = 0.0409, *z* = 0.807, *p* = 0.420). Even if saccades were generated randomly, we would observe more fixations on large objects than on small objects. Therefore, it is not surprising that young adults’ probability of fixating objects significantly increased with increasing object size (*b* = 1.028, *SE* = 0.0403, *z* = 25.485, *p* < 0.001). However, this effect was stronger for older adults, that is they fixated larger objects disproportionally more often than smaller objects (*b* = 0.1567, *SE* = 0.032, *z* = 4.901, *p* < 0.001). Importantly, object salience predicted gaze guidance above and beyond object size and eccentricity. Young adults fixated highly salient objects more frequently than objects with low visual salience (*b* = 0.3823, *SE* = 0.0384, *z* = 9.949, *p* < 0.001). Interestingly, this effect was significantly stronger for older adults (*b* = 0.0523, *SE* = 0.0219, *z* = 2.387, *p* = 0.017).

### Object-based fixation times

**Figure 5:**
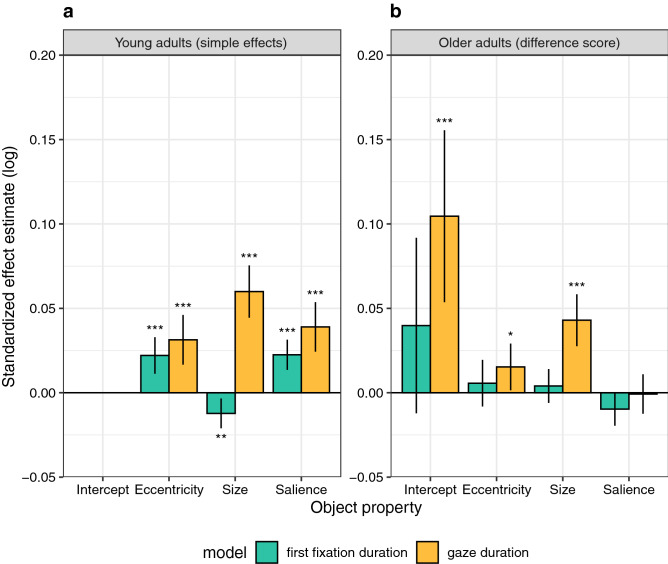
Fixed-effects results for linear mixed models fitting log-transformed fixation times for objects in scenes. One model evaluated first-fixation duration (green bars), the other one gaze duration (orange bars). Both models compared data for young and older adults. (**a**) Effects that were estimated for the young adults; the large coefficients for the intercept were not visualised (but see Table 3). (**b**) Difference scores, describing the difference between older and young adults. Error bars indicate 95% confidence intervals. Stars denote coefficients that were significantly different from zero (* |*t*|> 1.96, ** |*t*|> 2.576, *** |*t*|> 3.291).

According to the previous analysis, older adults had a reduced overall probability of fixating objects, without having a stronger central bias. Possibly, older adults engage longer with selected objects than young adults. To test this, we analysed two measures of fixation times. *First-fixation duration* is the duration of the initial fixation on the object, whereas *first-pass gaze duration* is the sum of all fixation durations from first entry to first exit^52,53^. Thus, gaze duration includes the duration of all immediate object refixations. The linear mixed model for each measure had the same fixed-effects structure as the object GLMM. Fixation times were log-transformed. The results are summarized in Table 3, and the fixed-effects estimates are visualized in Fig. 5.

**Table 3:** Fixation times for objects in scenes.

	First fixation duration			Gaze duration		
Fixed effects	*B*	*SE*	*t*	*B*	*SE*	*t*
Intercept: young	**5.4532**	**0.0181**	**301.878**	**5.609**	**0.0187**	**300.298**
Intercept: old–young	0.0398	0.0265	1.5	**0.1046**	**0.026**	**4.025**
Eccentricity: young	**0.0221**	**0.0055**	**4.003**	**0.0314**	**0.0075**	**4.176**
Eccentricity: old–young	0.0056	0.007	0.801	**0.0153**	**0.0071**	**2.165**
Size: young	**− 0.0122**	**0.0045**	**− 2.726**	**0.0599**	**0.0079**	**7.579**
Size: old–young	0.004	0.0051	0.781	**0.043**	**0.0078**	**5.478**
Salience: young	**0.0225**	**0.0046**	**4.933**	**0.039**	**0.0075**	**5.207**
Salience: old—young	**− **0.0097	0.005	**− **1.939	**− **0.0008	0.006	**− **0.128
**Random effects**						
Groups	Name		*SD*	Name		*SD*
Subject	Intercept		0.11310	Intercept		0.10987
	Object eccentricity		0.02193	Object eccentricity		0.01710
	Object size		0.00610	Object size		0.02234
	Object salience		0.00237	Object salience		0.00174
Object	Intercept		0.08463	Intercept		0.17556
Image	Intercept		0.02023	Intercept		0.03719

Significant coefficients are set in bold (|*t*|> 1.96). Results from two linear mixed models, fitting log-transformed first-fixation durations and gaze durations on objects. Standardized coefficients (*b*), standard errors (*SE*), and *t*-values for fixed effects and standard deviations (*SD*) for random effects are provided.

For young adults, all three object variables had significant effects on first-fixation durations. There was a significant effect of object eccentricity with longer first-fixation durations for more distant objects (*b* = 0.0221, *SE* = 0.0055, *t* = 4.003). Moreover, there was a significant negative effect of object size with shorter first-fixation durations for larger objects (*b* = − 0.0122, *SE* = 0.0045, *t* = − 2.726). Interestingly, there was also a significant positive effect of object salience with longer first-fixation durations for higher-salience objects (*b* = 0.0225, *SE* = 0.0046, *t* = 4.933). None of the interactions with age group were significant (Table 3), indicating that there was no evidence for significant differences between young and older adults for first-fixation durations.

Gaze durations for young adults were longer for more eccentric objects (*b* = 0.0314, *SE* = 0.0075, *t* = 4.176). They were also longer for larger objects and for higher-salience objects (object size: *b* = 0.0599, *SE* = 0.0079, *t* = 7.579, object salience: *b* = 0.039, *SE* = 0.0075, *t* = 5.207). The results for the intercept show that gaze durations were significantly longer for older adults compared with young adults (*b* = 0.1046, *SE* = 0.026, *t* = 4.025). Moreover, the effect of object eccentricity was significantly larger in older adults (*b* = 0.0153, *SE* = 0.0071, *t* = 2.165). The size effect was significantly larger in older adults too (*b* = 0.043, *SE* = 0.0078, *t* = 5.478), whereas the salience effect did not differ for young and older adults (*b* = − 0.0008, *SE* = 0.006, *t* = − 0.128).

### Distributions of within-object fixation locations: preferred viewing location

With the object GLMM we examined variables that affect whether objects are selected for fixation and found age-related differences in this regard. In addition, we analysed where viewers fixate within objects, once they are selected. Analyses considered initial fixations in first-pass viewing; that is, cases in which a saccade was launched from outside the object and led to a within-object fixation, irrespective of whether it was followed by an immediate refixation or not. Annotated objects differed in their sizes (width, height). Moreover, individual fixations differed with regard to the direction from which the eyes entered the object, though it has previously been demonstrated that most saccades enter the object from the left or from the right^5^. For analyses, landing positions within objects were normalized according to the size of the object^5,9^ and according to where the saccade originated^8^. These normalized *x*- and *y*-coordinates ranged from − 0.5 to 0.5, with 0 corresponding to fixations at the centre of the object and negative and positive values representing undershoots and overshoots of object centre, relative to the previous fixation location.

**Figure 6:**
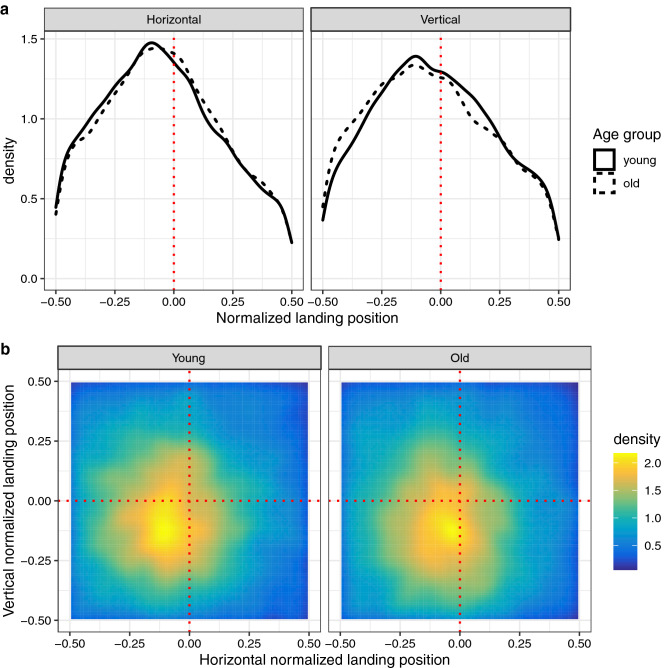
Preferred viewing location for objects in scenes. Analyses were based on initial fixations on objects. (**a**) Distributions of the horizontal (left panel) and vertical (right panel) components of normalized within-object landing positions are directly compared for young adults (solid line) and older adults (broken line). In both panels, the vertical red dotted line marks a landing position at the centre of the object. (**b**) Corresponding smoothed two-dimensional viewing location histograms for young adults (left panel) and older adults (right panel). The frequency information is displayed as variations in colour, with colours ranging from blue (few fixations) to yellow (many fixations) and passing through green and orange. The intersection of the two red dotted lines marks the centre of the object. See text for details on the normalization procedure.

The distributions of normalized landing positions are depicted in Fig. 6; for visualization purposes, the data were collapsed across all object sizes. First, the horizontal and vertical components of within-object fixation locations were considered separately, which allows for a direct comparison of densities for young and older adults (Fig. 6a). To accommodate the two-dimensional nature of the data, 2D density plots are additionally presented (Fig. 6b). The data revealed a peak (Fig. 6a) and/or a “hot spot” (Fig. 6b) close to the centre of the object, with a slight tendency to undershoot the centre. This PVL for objects in scenes was found for both young and older adults.

For statistical evaluation, two linear mixed models were specified, one each for horizontal and vertical normalized landing positions. Each model included the intercept, object size and their interactions with age group as fixed effects. As in the other object-based mixed models, object size was included as log-transformed object area, rather than including object width/height in the models testing normalized horizontal/vertical landing positions, respectively. The amplitude of the incoming saccade was not included as additional fixed effect because launch site, landing site and object centre do not in general fall on a single straight line. This would make the choice of an appropriate projection in 2D space a non-trivial endeavour, which is further complicated by the distortion of the objects’ aspect ratio when mapping it to the normalized coordinate frame used for representing the PVL.

The results are summarized in Table 4. For the reference group of young adults, the intercept was significantly smaller than 0 for both horizontal (*b* = − 0.0509, *SE* = 0.0051, *t* = − 9.952) and vertical landing positions (*b* = − 0.0294, *SE* = 0.0069, *t* = − 4.232). Due to the way landing positions were normalized, these results imply that the eyes tended to undershoot the centre of the object both horizontally as well as vertically. Interestingly, there were no significant differences between young and older adults (horizontal: *b* = 0.0102, *SE* = 0.007, *t* = 1.456, vertical: *b* = − 0.0109, *SE* = 0.0095, *t* = − 1.144). Moreover, there was a significant negative effect of object size such that larger objects were associated with smaller normalized landing positions, for both horizontal (*b* = − 0.0113, *SE* = 0.0027, *t* = − 4.238) and vertical dimensions (*b* = − 0.0173, *SE* = 0.0033, *t* = − 5.227). Thus, larger objects were associated with a stronger tendency to undershoot the centre of the object. Again, there was no evidence for significant differences between young and older adults (horizontal: *b* = − 0.0023, *SE* = 0.0027, *t* = − 0.861, vertical: *b* = 0.0053, *SE* = 0.0031, *t* = 1.728).

**Table 4:** Preferred viewing location for objects in scenes.

	Horizontal			Vertical		
Fixed effects	*B*	*SE*	*t*	*B*	*SE*	*t*
Intercept: young	**− 0.0509**	**0.0051**	**− 9.952**	**− 0.0294**	**0.0069**	**− 4.232**
Intercept: old–young	0.0102	0.007	1.456	**− **0.0109	0.0095	**− **1.144
Object size: young	**− 0.0113**	**0.0027**	**− 4.238**	**− 0.0173**	**0.0033**	**− 5.227**
Object size: old–young	**− **0.0023	0.0027	**− **0.861	0.0053	0.0031	1.728
**Random effects**						
Groups	Name		*SD*	Name		*SD*
Subject	Intercept		0.02793	Intercept		0.03965
	–		–	Object Size		0.00665
Object	Intercept		0.06035	Intercept		0.08033

Significant coefficients are set in bold (|*t*|> 1.96). Results from two separate linear mixed models, fitting horizontal and vertical landing positions for initial fixations on objects. Standardized coefficients (*b*), standard errors (*SE*), and *t*-values for fixed effects and standard deviations (*SD*) for random effects are provided.

### Effects of location-based visual salience on fixation selection

To compare effects of object-based and location-based visual salience, the object GLMM was complemented by a grid GLMM^48^. To this end, we applied an 8 × 6 grid such that each image and each AWS map was divided into 48 square patches. The grid GLMM allowed us to assess the effect of image salience across the entire scene, and without recurrence to objects. For this analysis approach, fixations were assigned to cells of an arbitrary grid rather than objects. Since all cells in the grid were of equal size, the GLMM did not include a fixed-effect for *size*. Otherwise, the fixed-effects structure for the grid GLMM was identical to the object GLMM.

The GLMM results are summarized in Table 5, and the fixed-effects estimates are visualized in Fig. 4 (blue bars in both panels). As before, the intercept in the GLMM represents the overall fixation probability, for which there was no significant difference between young and older observers (Table 5). On the probability scale, mean fixation probabilities were 0.220 and 0.224 for young and older adults, respectively. Reflecting the central bias, fixation probability was influenced by grid cell eccentricity. Specifically, young adults fixated distant cells less frequently than centrally located cells (*b* = − 0.5665, *SE* = 0.0364, *z* = − 15.564, *p* < 0.001), with no significant difference between age groups (*b* = 0.0424, *SE* = 0.0425, *z* = 0.996, *p* = 0.319). Importantly, cell salience influenced fixation probability beyond physical cell location in the scene in that cells with higher average AWS saliency were fixated more often (*b* = 0.6830, *SE* = 0.0289, *z* = 23.617, *p* < 0.001). As in the object GLMM, the effect of visual salience on fixation probability was differentially modulated by age, but now in the opposite direction. Thus, older adults showed a reduced effect of location-based visual salience (*b* = − 0.0399, *SE* = 0.0150, *z* = − 2.653, *p* = 0.008).

**Table 5:** Grid GLMM.

Fixed effects	*B*	*SE*	*z*	*p*
Intercept: young	− 1.2669	0.0333	− 38.084	< .001
Intercept: old–young	0.0223	0.0406	0.549	0.583
Central bias: young	− 0.5665	0.0364	− 15.564	< .001
Central bias: old–young	0.0424	0.0425	0.996	0.319
Salience: young	0.6830	0.0289	23.617	< .001
Salience: old–young	− 0.0399	0.0150	− 2.653	0.008
**Random effects**				
Groups	Name	Variance	Correlation	
Subject	Intercept	0.02995	Intercept	
	Central bias	0.03294	0.80	Central bias
	Salience	0.00323	0.32	0.32
Scene	Intercept	0.05510	Intercept	
	Central bias	0.07648	0.38	Central bias
	Salience	0.10889	− 0.32	0.30

Age-related effects on fixation probability during scene viewing are modelled with reference to equal-sized, square cells in a grid. Standardized coefficients (*b*), standard errors (*SE*), *z*- and *p*-values for fixed effects and variances and correlations for random effects are provided.

## Discussion

When inspecting images of real-world scenes, we move our eyes in a systematic manner. A key question regarding eye-movement control in scenes concerns the unit of saccadic selection. In principle, this selection can be based on localized features or on objects. A popular approach has been to extract various features at image locations and to investigate how these features drive the eyes in a bottom-up manner^54^. However, in recent years there have been a number of studies investigating the role of object-based selection^5,17,19^.

In the present study, we compared effects of location-based and object-based visual salience for young and older adults. The grid GLMM allowed us to assess the effect of image salience across the entire scene. For the sample of young adults, we had previously shown that location-based visual salience has an independent effect above and beyond what can be accounted for by the central fixation bias^49^. Here, we demonstrate that this effect is significantly *smaller* in older adults, which accords with previous research^45^. The object GLMM allowed us to test the hypothesis that visual salience aids prioritization among objects^19^. Both young and older adults selected highly salient objects more frequently for fixation than objects with low visual salience, while this effect was somewhat *larger* for older adults. In addition, we analysed where the first fixation on an object was placed within the object; a PVL close to the centre of the object^5^ was found for both young and older adults alike.

The question of what exactly an object is turns out to be less straightforward than our daily experience with objects may suggest. What constitutes an object depends on physical properties of the stimulus; however, it also depends on how we parse a scene in line with our behavioural goals^55^. Generally put, objects can be described as entities that can be individuated within a scene and potentially carry meaning. Object-based effects on eye guidance in scene perception have long been known to exist^56^. For example, research has shown how easily humans search for common objects in complex real-world scenes^57,58^. Moreover, a classic way to explore the influence of overall scene semantics on fixation selection has been to compare eye movements to objects that are either semantically consistent or inconsistent within a given scene context^53,59–61^.

Cognitive relevance theory is a theoretical account that emphasizes the importance of scene and object meaning^62,63^. In this view, the scene image is (only) needed to generate a visuospatial representation of potential saccade targets. Importantly, image features are thought to provide a flat (i.e., unranked) landscape of potential targets rather than a peaked salience map. Instead, potential saccade targets are ranked on the basis of relevance to the observer’s task and behavioural goals. The present results suggest that (object-based) visual salience *does* contribute to this ranking process, along with other variables, thereby challenging the assumption of a “flat” landscape of saccade targets.

Early presentations of the cognitive relevance account put emphasis on objects as saccade targets^5,63^. More recently, the cognitive relevance approach has been complemented by the meaning map approach^64,65^. Conceptually, a meaning map is analogous to a salience map, the difference being that it represents the spatial distribution of semantic rather than visual features. To create a meaning map, the image is divided into circular overlapping patches^64^. To measure the meaningfulness of these scene patches, human observers provide ratings which are then combined into a meaning map. Since meaning maps and salience maps are coded in the same format, researchers can assess the relative contributions of visual and semantic salience to fixation selection in scenes. The key result of several studies is that meaning as defined by meaning maps is more important for this selection process than visual salience^65^. It is important to note that the patches were presented independently of the scenes from which they were taken and independently of any task besides the rating itself. The correlation between such context-free meaning and visual salience is high^64^. Challenging the meaning map approach in its current form, results from a recent study suggest that meaning maps index the distribution of high-level visual features rather than meaning^66^.

The larger problem is that meaning can be defined in many ways^60^. Complicating things further, the meaning maps for young and older adults may be different. Young and older adults may also disagree on how meaningful or important an object is with respect to the global context of the scene. Therefore, we chose to remain agnostic about scene semantics but acknowledge that future work should take scene and object meaning into account. Being agnostic about semantics also provides one major rationale for using the AWS^20,67^ model rather than a more recent DNN-based model^68^. As the DNN-based models usually pre-train their lower layers on object classification tasks, it is likely that they carry some implicit semantic representation in these layers. In contrast, AWS implicitly carries information about objectness, but this is rather related to their “Gestalt” (in a broad sense) than to their meaning.

When dividing scene images into arrays of either circular patches^64^ or quadratic grid cells^48^ for data analysis reasons, researchers use procedures that are indifferent to an important theoretical question: What is the *unit* of the selection process? This is particularly evident in the context of meaning maps which, by design, decouple meaning from objects.

The object-based effects on fixation probability and analyses of within-object fixation locations reported here lend further support to the view that objects are important units of saccade targeting and, by inference, attentional selection in scene perception^5,11,17^. The results also allow for reconciling the salience view with the object view: although objects dominate over visual salience in selecting regions to be fixated^19^, objects themselves are prioritized by salience. Moreover, complementary analyses of fixation durations suggest that object salience did not only affect saccade target selection but also object encoding during fixation.

In previous studies, evidence for object-based selection in scenes was found for different task instructions^5,19^. In addition, studies in which a scene memorisation task was compared with an aesthetic preference judgement task have revealed only subtle differences in eye-movement behaviour^64,69–71^. Nevertheless, we cannot exclude the possibility that our object-related memory questions have biased participants toward fixating individual objects in the scenes. Moreover, it is known that effects of image salience differ for different tasks^72,73^. We leave it as a question for future research to determine whether the pattern of results reported here is modulated by task demands.

From a computer vision perspective, an apparent disadvantage of the object view, which it shares with the meaning map approach, is that it requires human annotators and/or raters. By contrast, both salience maps as well as proto-objects are image computable. Therefore, from a computer vision perspective it may be sufficient to establish correlations between salient locations and, for example, subjective interest points^74^, or to use proto-objects as proxy for objects^23^. However, from a cognitive science perspective, what constitutes the unit of selection during scene perception is an important question for theory building that should not be deferred for computational convenience.

In the present study, we also investigated age-related effects by comparing eye movements of young and older adults. Whereas there were no mean-level differences between young and older adults for number of fixations, fixation durations, and saccade amplitudes, the mixed-model analyses revealed systematic differences in viewing behaviour. The results from both grid and object GLMMs suggest that age does not modulate the central bias of fixation. This is in agreement with the previously reported finding that young and older adults show similar levels of explorative viewing behaviour overall^45^. The object-based results showed that, on average, observers fixated less than half of the annotated objects during the 6-s scene viewing period. Compared with young adults, older adults fixated significantly fewer of the annotated objects, but showed stronger effects of object size and salience on fixation probability.

In the GLMMs, the intercept represents the overall probability of fixating an object and/or a grid cell, and a smaller intercept should be associated with a larger central bias^48^. Why did we not observe this in the object GLMM? Object-based analyses of first-pass fixation times revealed that gaze durations, but not first-fixation durations, were longer for older adults than for young adults. This implies that older adults made more immediate object refixations than young adults. Thus, older adults engaged longer with selected objects than young adults, which may explain the finding that older adults fixated fewer of the annotated objects without exhibiting a stronger central bias.

Effects of visual salience on fixation probability showed an age-related dissociation: compared with young adults, older adults showed a reduced effect of location-based salience, but an increased effect of object-based salience. While a reduced effect of location-based visual salience is compatible with the idea that older adults rely more strongly on top-down as opposed to bottom-up control^45,75^, the object-based effects are less intuitive. Objects can be considered as high-level cues for selection. According to cognitive guidance models, selecting objects for fixation is a top-down process. Based on the present results, we argue that low-level variables like object size and salience also contribute to this selection process. Interestingly, older adults were more strongly guided by object size and salience than young adults.

The spatial analyses were complemented by temporal analyses related to objects that were fixated. In scene-perception research, gaze duration has been used as a measure of object encoding^53,59^. For young adults, gaze durations were longer for larger objects^76^. There was also an independent effect of object salience, with longer gaze durations for higher-salience objects. First-fixation durations were also modulated by object salience. In principle, the salience effects are consistent with previous location-based fixation-duration analyses^77,78^. Older adults showed a stronger effect of object size on gaze duration, whereas the effect of object salience did not differ between age groups.

Since the selection of the next fixation target is driven by information in parafoveal and peripheral vision^15,79^, effects of object size and salience may be associated with age-related changes in visual information processing. When arbitrary targets were embedded at 10° eccentricity in images of everyday scenes, older adults’ detection performance decreased with increasing age^80^. Moreover, using simple stimuli and fixation tasks it has been shown that older adults have a smaller useful field of view^81^ and are more susceptible to visual crowding^82^. However, whether and how these effects generalize to active scene perception is currently an open research question.

Research on scene perception has established a PVL for objects in scenes^5^. In previous research, the PVL was modulated by object size and launch site distance^11^, whereas it was unaffected by the lack of high-resolution information in central vision^15^. Therefore, in a sense, the PVL can be seen as a marker of extrafoveal processing abilities. The present data revealed a general tendency to undershoot the centre of objects, which was more pronounced for larger objects. The undershoot tendency observed for objects in scenes^5^ is consistent with findings from basic oculomotor research^12,13^. No differences between young and older adults were found. In future work, object width and height could be experimentally manipulated to investigate whether older adults are impaired in targeting objects that are particularly small or far away.

The present study focused on mean differences between young and older adults. Notably, the GLMM approaches used here also allow for investigating individual differences, by estimating variance/covariance components of subject-related random effects^49^. Hence, our method can readily be extended to the emerging question of individual differences in gaze behaviour when viewing naturalistic scenes^83,84^.

## Methods

### Participants

Analyses were based on data from a corpus of eye movements during scene viewing and sentence reading. Forty-two young adults who were students at the University of Edinburgh and 34 older adults from the community participated in the eye-tracking experiment. The young adults (8 men and 34 women) averaged 22.1 years of age (range = 18 years to 29 years), and the older adults (17 men and 17 women) averaged 72.1 years of age (range = 66 years to 83 years). All participants had normal or corrected-to-normal vision by self-report. Participants’ visual abilities were not independently assessed. Whereas this is a potential limitation of our study, meta-analytical results suggest that age-related differences in visual acuity do not moderate age-related differences in higher cognitive processing^85,86^. Participants gave written informed consent and received monetary compensation for their participation. The study was conducted in accordance with the Declaration of Helsinki and approved by the Psychology Research Ethics Committee of the University of Edinburgh.

The present analyses were based on the scene-viewing data. The data from the young adults were previously used to demonstrate how computational models of visual salience can be evaluated and compared by combining a-priori parcellation of scenes with GLMM^49^.

### Experimental setup and paradigm

Eye movements were recorded using an EyeLink 1000 Desktop mount system (SR-Research, Ottawa, ON, Canada). It was equipped with the 2000 Hz camera upgrade, allowing for binocular recordings at a sampling rate of 1000 Hz for each eye. Data from the right eye were analysed. The experiment was implemented with the SR Research Experiment Builder software.

Each participant viewed 150 colour photographs of real-world scenes (Fig. 1a), which were presented in random order. The scene images were displayed on a 21-inch CRT monitor at a screen resolution of 800 × 600 pixels (width × height). Head position and viewing distance were fixed at 90 cm from the screen using a chin rest. Accordingly, scenes subtended 25.78° × 19.34°. Before the onset of each scene, a central fixation check was performed. Afterwards, the scene was displayed for 6 s during which participants were free to move their eyes. To provide a common task across participants, they were informed that, on a given trial, they would view a real-life scene and that this may be followed by a question asking them to recall a specific detail of the scene. On 30 trials, a test question asking about the presence or absence of a particular object appeared after scene presentation to probe participants’ scene encoding.

### Evaluation of memory test performance

To evaluate participants’ responses to the memory test questions, we calculated signal detection theory measures^87^; that is, observers’ sensitivity (*d*’) and criterion (*c*). To avoid numerical issues with perfect false-alarm or hit rates, we applied the correction introduced by Hautus^88^ in all participants.

### Gaze data processing

Raw gaze data were converted into fixation sequence reports using the SR Research Data Viewer software. The initial, central fixation in a trial was excluded from all analyses. The last fixation in a trial happened when we removed the scene stimulus. The participant determined the location of the last fixation prior to the start of that fixation. Therefore, we included the last fixation in all analyses involving fixation positions, whereas we excluded it from analysis of fixation durations. Data processing was originally programmed in MATLAB (The MathWorks, Natick, MA, USA) and then re-implemented and generalized in Python (version 3.4; http://python.org) as an extension to our open-source toolbox GridFix (version 0.3; http://doi.org/10.5281/zenodo.4042996).

### Computation of salience maps and object properties

Salience maps were computed using the AWS model^20,67^. The AWS model relies on simple visual features, such as local colours and edge orientations, to predict fixations. In addition, it includes a statistical whitening procedure to improve performance. The AWS model was chosen because it performed well in previous model evaluations^4^ and because it was used in some of our previous studies^19,49^. The saliency maps from the AWS model were generated using the MATLAB code provided by the authors at http://persoal.citius.usc.es/xose.vidal/research/aws/AWSmodel.html. Parameters were kept at the authors’ default values, with the exception of the output scaling factor which was set to 1.0 instead of the default value of 0.5 to compute maps at full image resolution (Fig. 1c). By design of the AWS algorithm, maps are normalized to unit integral (i.e., the sum over all pixels equals 1).

**Figure 7:**
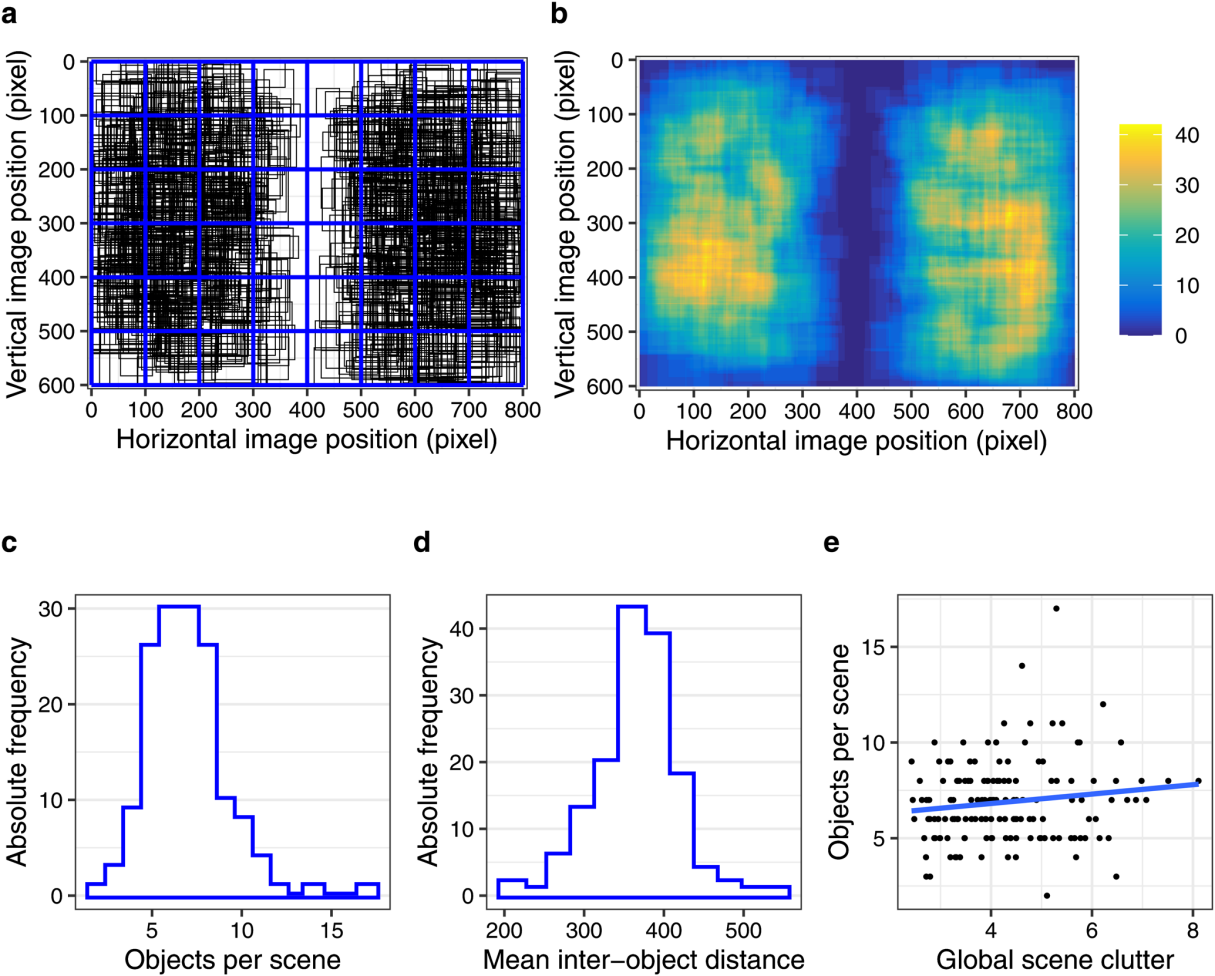
Objects in scenes. (**a**) Distribution and size of 1,032 object bounding boxes (black) across 150 scene images, with the scene grid (blue) overlaid as reference. (**b**) Pixel-based object map representing the distribution of all annotated objects across all scenes. (**c**) Histogram of the number of objects per scene. (**d**) Histogram of the mean inter-object distances per scene. (**e**) Scatter plot depicting the relationship between the number of annotated objects in a scene and the clutter associated with the scene; a linear regression line is added in blue. See text for details.

An independent annotator labelled objects in the scenes by providing object bounding boxes and object names using custom-made software. Whereas the bounding boxes were used for object-based eye-movement analyses, the names of the objects were used to construct the memory test questions. For a given object, a bounding box was drawn as the smallest possible rectangle encompassing the object (Fig. 1). The annotator was instructed to select objects that were of moderate size and were not occluded by other scene elements. Moreover, objects were chosen such that their spatial extension did not include the vertical midline of the scene. A total of 1032 objects were tagged across the 150 scene images. The mean width and height of annotated objects were 2.5° (*SD* = 1.4°) and 2.6° (*SD* = 1.5°), respectively. The mean Euclidean distance from object centre to scene centre was 8.6° (*SD* = 2.6°). Figure 7a shows the distribution and size of object bounding boxes across all scenes. Figure 7b additionally presents a summed object map. For a given image, all image pixel locations that belonged to an annotated object were coded with 1, whereas all other locations were coded with 0. These pixel-based maps were summed across all images to obtain a single object map. In Fig. 7b, this map is shown as heat map, with colours ranging from blue (no objects) to yellow (many objects).

To quantify the arrangement of objects within scenes, inter-object distances were determined. The distance between objects was calculated as the Euclidean distance between object centres (in pixels). For *k* objects in a scene, there are *k*(*k*-1)/2 unique distances between objects. Next, for each scene we computed the mean inter-object distance (Fig. 7d). For most images, the mean distances are of the order of 300 to 400 pixels, which indicates a fairly homogeneous spread of the annotated objects per scene.

The number of annotated objects varied across scenes (Fig. 7c); on average, there were 6.9 objects per scene (*SD* = 2.1). We did not aim at an exhaustive object annotation. To approximate the density of objects in the scene, we used the Feature Congestion measure of visual clutter^89^. For each image, a scalar representing the clutter of the entire image was computed, with larger values indicating more visual clutter. Whereas our scenes varied in clutter, there was no systematic relationship between the number of annotated objects in a scene and scene clutter (Fig. 7e).

Given that the AWS map values are normalized to sum to 1, the mean salience for each scene is 1/(800 × 600). The mean salience values for the annotated objects exceed the scene mean for 753 out of 1,032 objects (73%). A one-sample *t* test showed that object-based mean salience was significantly larger than scene-based mean salience, *t*(1031) = 21.37, *p* < 0.001. Moreover, we determined the maximum salience value for each scene and computed how often this maximum was part of an annotated object; this was the case for 39 out of 150 scenes (26%). Overall, this agrees with results from previous studies which showed that annotators tend to label “interesting” objects that coincide with salient locations in the image^17,90^. It is important to note that our objects showed appreciable variability in their salience (Fig. 3c).

Bounding boxes for each image were imported into GridFix and converted into binary masks, allowing for easy selection of all pixels corresponding to a bounding box in both images and salience maps. The GridFix toolbox also allows for using more fine-grained outlines of objects, such as tight object boundary polygons. When comparing the PVL for objects in scenes for annotations using polygons vs. boxes, Borji and Tanner^10^ found no qualitative differences. Therefore, we used object bounding boxes. Favouring conservative hypothesis testing, we refrained from adding a buffer around the object for data analysis^10^.

### Statistical analysis

Statistical analyses were conducted using the R system for statistical computing (version 3.6.0; R Core Team 2019). (G)LMM were fit to the data using the (g)lmer programme of the lme4 package^91^ (version 1.1–21), with the bobyqa optimizer (lmer) or a combination of Nelder-Mead and bobyqa (glmer). GLMMs were fit by Laplace approximation. LMMs were estimated using the restricted maximum likelihood (REML) criterion, which is the default model-fitting approach.

Fixation probability was measured by a binary response variable: for a given observer and image, we coded whether a given object and/or grid cell was fixated (1) or not fixated (0). The data were analysed with binomial GLMMs for which we used the logit link function as the default for glmer. In binomial logit mixed models, the parameter estimates are obtained on the log-odds or logit scale, and thus represent the log odds of selecting a particular competing object or grid cell^92^.

Data were modelled at the level of individual observations. For grid-based analyses, the number of matrix entries is determined by the number of participants × number of scene images × (number of grid cells − 1); the grid cell on which the very first fixation fell was excluded^48,49^. The GridFix toolbox creates the observation matrix based on trials for which fixation data are available. Given that there were 10 missing trials in the data set, the observation matrix contained 535,330 rows for the grid-based analyses. For the object-based analyses of fixation probability, the number of entries in the observation matrix is determined by the number of participants × number of annotated objects. For object-based analyses of fixation times and landing positions, the data matrix was reduced to fixated objects only. If the first visit to an object included the last fixation in a trial, this object was excluded from the analysis of fixation times. Cases in which the initial, central fixation coincided with the first fixation on an object were excluded from all object-based analyses (*N* = 15).

LMMs were used for analysing continuous response variables, specifically measures of fixation time and of horizontal and vertical normalized within-object landing positions.

For the (G)LMMs we report regression coefficients (*b*) and their standard errors (*SE*) along with the corresponding *z*-values (GLMM: *z* = *b*/*SE*) or *t*-values (LMM: *t* = *b*/*SE*). For GLMMs, *p*-values based on asymptotic Wald tests are additionally provided. For LMMs, a two-tailed criterion (|*t*|> 1.96) was used to determine significance at the alpha level of 0.05^93^.

A mixed-effects model contains both fixed-effects and random-effects terms. For the fixed-effects structure, three stimulus-related input variables were considered. To account for observers’ central bias of fixation, an anisotropic Euclidean central-bias predictor was included^49^. To this end, the distance between the centre of each object and/or grid cell to the centre of the scene was determined, whereby vertical distances were scaled by a factor of 0.45. The scaling factor was applied because fixation positions in scene viewing typically show a greater spread of fixations horizontally than vertically^94^. Visual salience was defined as the mean over the saliency map’s values within the object’s bounding box and/or grid cell. For object-based analyses, object size was defined as the log-transformed area of the object’s bounding box. Stimulus-related input variables were measured within participants on a continuous scale. For the (G)LMM analyses, they were centred and scaled (*z*-transformed). Age group is a categorical variable. Moreover, it is a between-participants factor, since each participant can only belong to one age group. To include age group as predictor in the statistical models, we used treatment coding (aka dummy coding) with the group of young adults as the reference category. Age-related differences were tested through interactions between “age group” and a given continuous predictor. To give an example, for the fixed effect of visual salience the GLMM will first test the effect of object-based and/or grid-based salience on fixation probability for the young adults (simple effect). In addition, the GLMM will test whether this effect was significantly different for the group of older adults (interaction). The actual coefficient for the effect of salience in older adults can be derived by summing the simple effect coefficient and the interaction coefficient.

Inclusion of random factors allows for estimating the extent to which mean responses vary across levels of the random factor. Detailed considerations regarding the inclusion of random factors are provided in our previous publication^49^. For a given type of analysis, the random-effects structure of the mixed model was determined according to the study design and underlying theory. In our data, the random factor “subject” is naturally nested under “age group”. The random factor “object” was nested under the random factor “scene”. Subjects and scenes were crossed. All (G)LMMs were set up to include the “maximal” structure^95^ for the random factor “subject”. Thus, a by-subject random intercept was included along with all possible random slopes and correlation parameters. This way, we acknowledge that subjects may differ in their responses above and beyond belonging to their age group, which was modelled as a fixed effect. For object-based analyses, an additional random intercept for objects nested within scenes was included. By-object random slopes were not included as each individual object had exactly one eccentricity, one size and one salience value. Compared with objects, grid cells are arbitrarily chosen units. Therefore, the grid GLMM did not include a random intercept for grid cell. However, all models were designed to include a by-scene intercept. In the object GLMM, by-scene random slopes were not included as there were no compelling reasons to expect additional by-scene varying effects of object-based variables. In the grid GLMM, by-scene random slopes for central bias and salience as well as the correlation parameters were included, consistent with our previous work^49^.

Using Wilkinson notation^96^, the model formula for the grid GLMM was:

1 $$\text{Fixated} \sim 1+\text{Age}+\text{CentralBias}+\text{CentralBias:Age}+\text{Salience}+\text{Salience:Age}+(1+\text{CentralBias}+\text{Salience}|\text{Subject})+(1+\text{CentralBias}+\text{Salience}|\text{Scene})$$

The model formula for the object-based mixed models fitting either fixation probability or fixation times was:

2 $$\text{Response variable} \sim 1+\text{Age}+\text{CentralBias}+\text{CentralBias:Age}+\text{Size}+\text{Size:Age}+\text{Salience}+\text{Salience:Age}+(1+\text{CentralBias}+\text{Size}+\text{Salience}|\text{Subject})+(1|\text{Object})+(1|\text{Scene})$$

For the analysis of within-object landing positions, object size was the only object-related input variable. The random-effects structure of these LMMs was further simplified in a stepwise manner by removing random effects for which the estimated variances were particularly small (see Table 4 for the final models that were supported by the data).

Figures 2, 3, 4, 5, 6, 7 were created with the ggplot2 package^97^ (version 3.2.1) supplied in R. The smoothed density estimates in Fig. 2 and Fig. 6a were created with the geom_density function. The smoothed two-dimensional density estimates in Fig. 6b were created with the stat_density2d function, which uses the kde2d function from the MASS package; the normal reference bandwidth was used.

## Data Availability

The datasets analysed during the current study are available from the corresponding author on reasonable request. The updated open-source Python toolbox GridFix is available at https://github.com/ischtz/gridfix.

## References

[1] Koch C, Ullman S (1985). Shifts in selective visual attention: Towards the underlying neural circuitry. Hum. Neurobiol..

[2] Itti L, Koch C, Niebur E (1998). A model of saliency-based visual attention for rapid scene analysis. IEEE Trans. Pattern Anal. Mach. Intell..

[3] Parkhurst D, Law K, Niebur E (2002). Modeling the role of salience in the allocation of overt visual attention. Vision. Res..

[4] Borji A, Sihite DN, Itti L (2013). Quantitative analysis of human-model agreement in visual saliency modeling: A comparative study. IEEE Trans. Image Process..

[5] Nuthmann A, Henderson JM (2010). Object-based attentional selection in scene viewing. J. Vis..

[6] Dziemianko M, Keller F (2013). Memory modulated saliency: A computational model of the incremental learning of target locations in visual search. Vis. Cogn..

[7] Xu J, Jiang M, Wang S, Kankanhalli MS, Zhao Q (2014). Predicting human gaze beyond pixels. J. Vis.

[8] Foulsham T, Kingstone A (2013). Optimal and preferred eye landing positions in objects and scenes. Q. J. Exp. Psychol..

[9] Anderson NC, Donk M (2017). Salient object changes influence overt attentional prioritization and object-based targeting in natural scenes. PLoS ONE.

[10] Borji A, Tanner J (2016). Reconciling saliency and object center-bias hypotheses in explaining free-viewing fixations. IEEE Trans. Neural Netw. Learn. Syst..

[11] Pajak M, Nuthmann A (2013). Object-based saccadic selection during scene perception: Evidence from viewing position effects. J. Vis..

[12] Becker W, Fuchs AF (1969). Further properties of the human saccadic system: Eye movements and correction saccades with and without visual fixation points. Vis. Res..

[13] Abrams RA, Meyer DE, Kornblum S (1989). Speed and accuracy of saccadic eye movements: Characteristics of impulse variability in the oculomotor system. J. Exp. Psychol. Hum. Percept. Perform..

[14] Yun K, Peng Y, Samaras D, Zelinsky GJ, Berg TL (2013). Exploring the role of gaze behavior and object detection in scene understanding. Front. Psychol..

[15] Nuthmann A (2014). How do the regions of the visual field contribute to object search in real-world scenes? Evidence from eye movements. J. Exp. Psychol. Hum. Percept. Perform..

[16] Itti L, Koch C (2000). A saliency-based search mechanism for overt and covert shifts of visual attention. Vis. Res..

[17] Einhäuser W, Spain M, Perona P (2008). Objects predict fixations better than early saliency. J. Vis..

[18] Borji A, Sihite DN, Itti L (2013). Objects do not predict fixations better than early saliency: A re-analysis of Einhäuser et al.’s data. J. Vis..

[19] Stoll J, Thrun M, Nuthmann A, Einhäuser W (2015). Overt attention in natural scenes: Objects dominate features. Vis. Res..

[20] Garcia-Diaz A, Leborán C, Fdez-Vidal XR, Pardo XM (2012). On the relationship between optical variability, visual saliency, and eye fixations: A computational approach. J. Vis..

[21] LeCun Y, Bengio Y, Hinton G (2015). Deep learning. Nature.

[22] Kümmerer, M., Wallis, T. S. A., Gatys, L. A. & Bethge, M. Understanding low- and high-level contributions to fixation prediction. In *IEEE Int. Conf. Comput. Vis. (ICCV)* 4799–4808. 10.1109/iccv.2017.513 (2017).

[23] Chen Y, Zelinsky GJ (2019). Is there a shape to the attention spotlight? Computing saliency over proto-objects predicts fixations during scene viewing. J. Exp. Psychol. Hum. Percept. Perform..

[24] Russell AF, Mihalas S, von der Heydt R, Niebur E, Etienne-Cummings R (2014). A model of proto-object based saliency. Vis. Res..

[25] Walther D, Koch C (2006). Modeling attention to salient proto-objects. Neural Netw..

[26] Henrich J, Heine SJ, Norenzayan A (2010). The weirdest people in the world?. Behav. Brain Sci..

[27] Erel H, Levy DA (2016). Orienting of visual attention in aging. Neurosci. Biobehav. Rev..

[28] Owsley C (2011). Aging and vision. Vis. Res..

[29] Salthouse TA (2010). Selective review of cognitive aging. J. Int. Neuropsychol. Soc..

[30] Owsley C, Sekuler R, Siemsen D (1983). Contrast sensitivity throughout adulthood. Vis. Res..

[31] Elliott D, Whitaker D, MacVeigh D (1990). Neural contribution to spatiotemporal contrast sensitivity decline in healthy ageing eyes. Vis. Res..

[32] Jaffe GJ, Alvarado JA, Juster RP (1986). Age-related changes of the normal visual field. Arch. Ophthalmol..

[33] Theeuwes J (2010). Top-down and bottom-up control of visual selection. Acta Psychol..

[34] Kramer AF, Hahn S, Irwin DE, Theeuwes J (2000). Age differences in the control of looking behavior: Do you know where your eyes have been?. Psychol. Sci..

[35] Ridderinkhof KR, Wijnen JG (2011). More than meets the eye: Age differences in the capture and suppression of oculomotor action. Front. Psychol..

[36] Kramer AF, Hahn S, Irwin DE, Theeuwes J (1999). Attentional capture and aging: Implications for visual search performance and oculomotor control. Psychol. Aging.

[37] Irving EL, Steinbach MJ, Lillakas L, Babu RJ, Hutchings N (2006). Horizontal saccade dynamics across the human life span. Invest. Ophthalmol. Vis. Sci..

[38] Pitt MC, Rawles JM (1988). The effect of age on saccadic latency and velocity. Neuro-Ophthalmol..

[39] Warabi T, Kase M, Kato T (1984). Effect of aging on the accuracy of visually guided saccadic eye movement. Ann. Neurol..

[40] Sharpe JA, Zackon DH (1987). Senescent saccades: effects of aging on their accuracy, latency and velocity. Acta Oto-Laryngol..

[41] Warren DE, Thurtell MJ, Carroll JN, Wall M (2013). Perimetric evaluation of saccadic latency, saccadic accuracy, and visual threshold for peripheral visual stimuli in young compared with older adults. Invest. Ophthalmol. Vis. Sci..

[42] Paterson KB (2020). Effects of normative aging on eye movements during reading. Vision.

[43] Kliegl R, Grabner E, Rolfs M, Engbert R (2004). Length, frequency, and predictability effects of words on eye movements in reading. Eur. J. Cognit. Psychol..

[44] Rayner K, Reichle ED, Stroud MJ, Williams CC, Pollatsek A (2006). The effect of word frequency, word predictability, and font difficulty on the eye movements of young and older readers. Psychol. Aging.

[45] Açik A, Sarwary A, Schultze-Kraft R, Onat S, König P (2010). Developmental changes in natural viewing behavior: Bottom-up and top-down differences between children, young adults and older adults. Front. Psychol..

[46] Helo A, Pannasch S, Sirri L, Rämä P (2014). The maturation of eye movement behavior: Scene viewing characteristics in children and adults. Vis. Res..

[47] van Renswoude DR, Visser I, Raijmakers MEJ, Tsang T, Johnson SP (2019). Real-world scene perception in infants: What factors guide attention allocation?. Infancy.

[48] Nuthmann A, Einhäuser W (2015). A new approach to modeling the influence of image features on fixation selection in scenes. Ann. NY Acad. Sci..

[49] Nuthmann A, Einhäuser W, Schütz I (2017). How well can saliency models predict fixation selection in scenes beyond central bias? A new approach to model evaluation using generalized linear mixed models. Front. Hum. Neurosci..

[50] Mannan SK, Ruddock KH, Wooding DS (1996). The relationship between the locations of spatial features and those of fixations made during visual examination of briefly presented images. Spat. Vis..

[51] Tatler BW (2007). The central fixation bias in scene viewing: Selecting an optimal viewing position independently of motor biases and image feature distributions. J. Vis..

[52] Rayner K (1998). Eye movements in reading and information processing: 20 years of research. Psychol. Bull..

[53] Henderson JM, Weeks PA, Hollingworth A (1999). The effects of semantic consistency on eye movements during complex scene viewing. J. Exp. Psychol. Hum. Percept. Perform..

[54] Borji A, Itti L (2013). State-of-the-art in visual attention modeling. IEEE Trans. Pattern Anal. Mach. Intell..

[55] Chen Z (2012). Object-based attention: A tutorial review. Atten. Percept. Psychophys..

[56] Belardinelli A, Mancas M, Ferrera VP, Riche N, Taylor JG (2016). Object-based attention: Cognitive and computational perspectives. From Human Attention to Computational Attention: A Multidisciplinary Approach.

[57] Biederman I, Glass AL, Stacy EW (1973). Searching for objects in real-world scenes. J. Exp. Psychol..

[58] Malcolm GL, Henderson JM (2010). Combining top-down processes to guide eye movements during real-world scene search. J. Vis..

[59] Loftus GR, Mackworth NH (1978). Cognitive determinants of fixation location during picture viewing. J. Exp. Psychol. Hum. Percept. Perform..

[60] Spotorno S, Tatler BW (2017). The elephant in the room: Inconsistency in scene viewing and representation. J. Exp. Psychol. Hum. Percept. Perform..

[61] Coco MI, Nuthmann A, Dimigen O (2020). Fixation-related brain potentials during semantic integration of object-scene information. J. Cognit. Neurosci..

[62] Henderson JM, Brockmole JR, Castelhano MS, Mack M, van Gompel RPG, Fischer MH, Murray WS, Hill RL (2007). Visual saliency does not account for eye movements during visual search in real-world scenes. Eye Movements: A Window on Mind and Brain.

[63] Henderson JM, Malcolm GL, Schandl C (2009). Searching in the dark: Cognitive relevance drives attention in real-world scenes. Psychon. Bull. Rev..

[64] Henderson JM, Hayes TR (2017). Meaning-based guidance of attention in scenes as revealed by meaning maps. Nat. Hum. Behav..

[65] Henderson JM, Hayes TR, Peacock CE, Rehrig G (2019). Meaning and attentional guidance in scenes: A review of the meaning map approach. Vision.

[66] Pedziwiatr, M. A., Kümmerer, M., Wallis, T. S. A., Bethge, M. & Teufel, C. Meaning maps and saliency models based on deep convolutional neural networks are insensitive to image meaning when predicting human fixations. *Cognition* **206**, 104465. 10.1016/j.cognition.2020.104465 (2021). 10.1016/j.cognition.2020.104465 33096374

[67] Garcia-Diaz A, Fdez-Vidal XR, Pardo XM, Dosil R (2012). Saliency from hierarchical adaptation through decorrelation and variance normalization. Image Vis. Comput..

[68] Kümmerer, M., Wallis, T. S. A. & Bethge, M. DeepGaze II: Reading fixations from deep features trained on object recognition. *arXiv*. https://arxiv.org/abs/1610.01563 (2016).

[69] Einhäuser W, Nuthmann A (2016). Salient in space, salient in time: Fixation probability predicts fixation duration during natural scene viewing. J. Vis..

[70] Nuthmann A (2017). Fixation durations in scene viewing: Modeling the effects of local image features, oculomotor parameters, and task. Psychon. Bull. Rev..

[71] Cronin DA, Hall EH, Goold JE, Hayes TR, Henderson JM (2020). Eye movements in real-world scene photographs: General characteristics and effects of viewing task. Front. Psychol..

[72] Koehler K, Guo F, Zhang S, Eckstein MP (2014). What do saliency models predict?. J. Vis..

[73] Rahman S, Bruce N (2015). Visual saliency prediction and evaluation across different perceptual tasks. PLoS ONE.

[74] Masciocchi CM, Mihalas S, Parkhurst D, Niebur E (2009). Everyone knows what is interesting: Salient locations which should be fixated. J. Vis..

[75] Madden DJ (2007). Aging and visual attention. Curr. Dir. Psychol. Sci..

[76] Wang H-C, Hwang AD, Pomplun M (2010). Object frequency and predictability effects on eye fixation durations in real-world scene viewing. J. Eye Mov. Res..

[77] Tatler BW, Brockmole JR, Carpenter RHS (2017). LATEST: A model of saccadic decisions in space and time. Psychol. Rev..

[78] Mathôt S, Siebold A, Donk M, Vitu F (2015). Large pupils predict goal-driven eye movements. J. Exp. Psychol. Gen..

[79] Einhäuser W, Atzert C, Nuthmann A (2020). Fixation durations in natural scene viewing are guided by peripheral scene content. J. Vis..

[80] Gruber N (2014). Effects of age and eccentricity on visual target detection. Front. Aging Neurosci..

[81] Ball KK, Beard BL, Roenker DL, Miller RL, Griggs DS (1988). Age and visual search: Expanding the useful field of view. J. Opt. Soc. Am. A-Opt. Image Sci. Vis..

[82] Scialfa CT, Cordazzo S, Bubric K, Lyon J (2013). Aging and visual crowding. J. Gerontol. Ser. B-Psychol. Sci. Soc. Sci..

[83] Li A, Chen Z (2018). Personalized visual saliency: Individuality affects image perception. IEEE Access.

[84] de Haas B, Iakovidis AL, Schwarzkopf DS, Gegenfurtner KR (2019). Individual differences in visual salience vary along semantic dimensions. Proc. Natl. Acad. Sci. USA.

[85] Houston JR, Bennett IJ, Allen PA, Madden DJ (2016). Visual acuity does not moderate effect sizes of higher-level cognitive tasks. Exp. Aging Res..

[86] La Fleur CG, Salthouse TA (2014). Out of sight, out of mind? Relations between visual acuity and cognition. Psychon. Bull. Rev..

[87] Stanislaw H, Todorov N (1999). Calculation of signal detection theory measures. Behav. Res. Methods Instr. Comput..

[88] Hautus MJ (1995). Corrections for extreme proportions and their biasing effects on estimated values of d'. Behav. Res. Methods Instr. Comput..

[89] Rosenholtz R, Li Y, Nakano L (2007). Measuring visual clutter. J. Vis..

[90] Elazary L, Itti L (2008). Interesting objects are visually salient. J. Vis..

[91] Bates DM, Mächler M, Bolker BM, Walker S (2015). Fitting linear mixed-effects models using lme4. J. Stat. Softw..

[92] Barr DJ (2008). Analyzing 'visual world' eyetracking data using multilevel logistic regression. J. Mem. Lang..

[93] Baayen RH, Davidson DJ, Bates DM (2008). Mixed-effects modeling with crossed random effects for subjects and items. J. Mem. Lang..

[94] Clarke ADF, Tatler BW (2014). Deriving an appropriate baseline for describing fixation behaviour. Vis. Res..

[95] Barr DJ, Levy R, Scheepers C, Tily HJ (2013). Random effects structure for confirmatory hypothesis testing: Keep it maximal. J. Mem. Lang..

[96] Wilkinson GN, Rogers CE (1973). Symbolic description of factorial models for analysis of variance. R. Stat. Soc. Ser. C-Appl. Stat..

[97] Wickham H (2016). ggplot2: Elegant graphics for data analysis.

